# Concentration of Serum Matrix Metalloproteinase-3 in Patients With Primary Biliary Cholangitis

**DOI:** 10.3389/fimmu.2022.885229

**Published:** 2022-04-22

**Authors:** Alicja Bauer, Andrzej Habior

**Affiliations:** ^1^ Department of Biochemistry and Molecular Biology, Centre of Postgraduate Medical Education, Warsaw, Poland; ^2^ Department of Gastroenterology, Hepatology and Clinical Oncology Centre of Postgraduate Medical Education, Warsaw, Poland; ^3^ Clinic of Polish Gastroenterology Foundation, Warsaw, Poland

**Keywords:** primary biliary cholangitis, liver fibrosis, autoantibodies, metalloproteinases, MMP-3

## Abstract

**Background and Aims:**

Metalloproteinases (MMPs) are involved in many distinct processes in the liver. Matrix metalloproteinase-3 (MMP-3) plays an important role in connective tissue remodeling, degradation of collagen (types II, III, IV, IX, and X), proteoglycans, fibronectin, laminin, and elastin. In addition, MMP-3 can also activate other MMPs such as MMP-1, MMP-7, and MMP-9. Primary biliary cholangitis (PBC) is a cholestatic, autoimmune liver disease, characterized by the progressive destruction of intrahepatic bile ducts, leading to cholestasis, fibrosis, cirrhosis, and liver failure. Fibrosis is the result of an imbalance between production and degradation of the extracellular matrix surrounding hepatocytes. Our aim in the present study was to determine whether the measurement of serum MMP-3 is clinically useful for assessing ongoing liver fibrosis in patients with PBC.

**Methods:**

The MMP-3 concentration was determined in 182 PBC patients and 80 non-PBC controls using a commercially available ELISA kit.

**Results:**

Higher concentrations of MMP-3 were found in 61% of PBC patients. PBC subjects had greater MMP-3 levels than controls: 68.9 ± 62.6 vs 21.3 ± 7.4 ng/mL, p < 0.001 for healthy subjects; 68.9 ± 62.6 vs 22.7 ± 7.6 ng/mL, p = 0.022 for autoimmune hepatitis controls; and 68.9 ± 62.6 vs 37.2 ± 17.4 ng/mL, p = 0.002 for primary sclerosing cholangitis controls. The serum MMP-3 concentration was significantly elevated in patients with higher bilirubin concentration (107.6 ± 85.8 vs 61.6 ± 46.1 ng/mL, p < 0.001) and was correlated with the level of antimitochondrial antibodies specific for PBC. The concentration of MMP-3 in sera of PBC patients was also found to correlate with the state of liver fibrosis (OR = 4.3; p < 0.01).

**Conclusions:**

Our study demonstrated significantly higher MMP-3 levels in PBC patients than in healthy and pathological controls. Increased MMP-3 concentrations were positively correlated with various clinical and immunological parameters, and advanced liver fibrosis. The level of MMP-3 was associated with hepatic dysfunction and could play a role in the pathophysiology of hepatic fibrosis in PBC.

## Introduction

Primary biliary cholangitis (PBC) is an autoimmune chronic progressive cholestatic liver disease. PBC is characterized by infiltration of lymphocytes and plasma cells into the bile duct and, as a result of inflammation and destruction, leads to fibrosis and cirrhosis of the liver (1–7). The most characteristic immunological features of the disease are antimitochondrial antibodies (AMAs) (8–10). The M2 fraction of AMAs, directed against the 2-oxoacid-dehydrogenase complex of the inner mitochondrial membrane, is detected in up to 95% of PBC patients. In addition, approximately 50% of PBC patients have different types of antinuclear antibodies (ANAs) (11–18). Several nuclear structures are considered as targets for the antinuclear antibodies ANA) at the PBC. The detection of two antinuclear immunofluorescence patterns (“Rim-like/membranous” and “Multiple nuclear dots”) display very high diagnostic accuracy for PBC diagnosis as previously demonstrated (19). Antibodies giving these patterns recognize the gp210 nuclear pore membrane protein and the sp100 nuclear body protein, respectively. ANA against nuclear envelope proteins such as anti-gp210 antibodies are not common but highly specific for PBC. PBC-specific MND pattern produces antibodies directed against the structural components of the nuclear protein of promyelocytic leukemia body - NB PML. Among the numerous substructures of the nucleus of the NB PML the proteins PML and Sp100 appear to be the most important.

Matrix metalloproteinases (MMPs) are a 28-membered family of zinc-dependent endopeptidases. They are the main enzymes that degrade extracellular matrix proteins and play an important role in the process of tissue remodeling and repair under physiological and pathological conditions (20–22). They are produced by most normal cells, including mast cells, osteoblasts, odontoblasts, dendritic cells, microglial cells, smooth myocytes, keratinocytes and endothelial cells. These enzymes are also secreted by inflammatory cells, macrophages and T lymphocytes. The MMP family consists of four major subtypes: collagenases, gelatinases, stromelysins and membrane-type metalloproteinases (23).

MMPs are involved in many distinct physiological processes in the liver. They are also critical in the pathogenicity and progression of liver diseases, including fibrosis cirrhosis and cancer, however the mechanisms underlying this phenomenon are largely unknown (24, 25). The pathobiology of liver disease is associated with dysregulated remodeling of the extracellular matrix (ECM) and its excessive production. As the catalytic activity of MMPs is specific to collagen-like peptides, this may result in neoplastic metastases and fibrosis (26–28). MMPs have been also described as biomarkers in various liver diseases (29–33). Some research groups have proposed to recognize circulating serum MMPs as specified reliable biomarkers of active fibrosis (34–40).

It has been reported that the altered expression of MMP-3 is linked with liver inflammation and fibrosis (41). MMP-3 is a stromelysin that is expressed in a variety of cell types, including hepatocellular carcinoma cells and hepatic stellate cells (42–44). MMP-3 is also associated with the excretion of protein ectodomains from the cell surface and degradation of extracellular matrix substrates, including collagens (type III, IV and V) and non-collagen ECM components such as laminins, fibronectin, osteopontin and proteoglycans (45). MMP-3 is involved in the release of chemotactic cytokines that initiate macrophage and leukocyte infiltration, and activate the tumor necrosis factor. MMP-3 can also stimulate other MMPs such as MMP-1, MMP-7, and MMP-9 (46, 47).

The polymorphism of MMP-3 (genetic variants) is associated with poor prognosis in hepatocellular carcinoma and primary sclerosing cholangitis (PSC) (47–50).

The aim of the study was to determine whether the measurement of serum MMP-3 is clinically useful for assessing ongoing liver fibrosis in patients with PBC and assess whether an increased MMP-3 concentration may be associated with biochemical parameters, primarily with the level of bilirubin, and presence of specific antibodies.

## Materials and Methods

### Patients

Serum samples were collected from 182 patients (176 women, 6 men; median age: 50; range: 27–75 years), diagnosed at the Centre of Postgraduate Medical Education (Warsaw, Poland). The diagnosis of PBC was established using generally accepted criteria, according to the practical guidelines of the European Association For The Study of Liver Diseases (EASL) for PBC (51, 52). A biopsy was performed in all subjects. In AMA -negative PBC patients diagnosis was supported by antinuclear antibody positivity or by liver biopsy. We excluded patients with serum levels positive for the hepatitis B surface antigen (HBsAg), anti-hepatitis A (IgM) and hepatitis C virus, patients with alcoholism and AIH (autoimmune hepatitis)/PBC overlap syndrome. In most patients, the diagnosis was made within one year after the onset of symptoms. The main measurements were time of death from liver failure or time to liver transplantation. The pathologic control group consisted of: 40 patients (16 females, 24 males; median age: 48; age range: 20-65 years) with PSC; 10 patients with AIH (9 females, 1 male; median age: 48; age range: 22-68 years). Serum samples from 30 healthy adult blood donors (22 females, 8 males; median age: 33; age range: 19-53 years) were collected at the Warsaw Blood Bank.

The study protocol was conducted in accordance with the ethical guidelines of the Declaration of Helsinki and was approved by the Ethical Committee of the Centre of Postgraduate Medical Education, Warsaw (approval number 71/PB/2019).

### Detection of MMP-3

The MMP-3 concentration was assessed using a commercially available ELISA kit AESKULISA^®^ MMP-3 (AESKU, Wendelsheim, Germany), according to the manufacturer’s instructions. Intra-assay and inter-assay performances were 3.4% and 4.6%, respectively. MMP-3 concentrations > 30 ng/mL were considered positive.

### Detection of AMA M2 Autoantibodies

AMAs type M2 were determined using the commercially available kit QUANTA Lite^®^ M2 EP- MIT3 ELISA (Inova Diagnostics, San Diego, CA, USA), according to the manufacturer’s instructions.

### Statistical Analysis

Prevalence rates were compared between groups using the chi-square test and Fisher’s exact test. Continuous data were summarized as mean ± standard deviation (SD), and categorical data were summarized as frequencies. Continuous variables were evaluated using the Mann-Whitney test and were expressed as median ± interquartile range (IQR). P < 0.05 was considered statistically significant. All statistical analyses were performed using the Statistica 8.0 software (Stat-Soft, Cracow, Poland) and MedCal for Windows, version 7.4.1.0 (MedCal Software, Mariakerke, Belgium).

## Results

### Clinical, Histological and Laboratory Features of PBC Patients and Control Groups

The clinical, histological and laboratory features of PBC patients are presented in **Table 1**. The mean age at PBC diagnosis was 50 years and 176 patients out of 182 were female. Over 50% of patients had elevated total bilirubin levels. In over 70% of the tested samples increased activity of AP, g-GT, AST and ALT was found, and 172 (86%) patients were positive for AMA M2. Liver biopsies were obtained in all patients: stages I or II were observed in 119 subjects, while stages III or IV were found in 58 cases.

**Table 1:** The demographic, biochemical, immunological and histological features of PBC patients and control groups.

	PBC patients (n=182)	AIH patients (n=10)	PSC patients (n=40)	Healthy adult blood donors (n = 30)
Age, years	50 (27-75)	48 (22 -68)	48 (20-65)	33 (19 – 53)
Females/males	176/6	9/1	16/24	22/8
Bilirubin (Total), mg/dL	1.9 (1.9)	1.8 (2.1)	1.5 (2.5)	0.7 (0.5)
AST, U/L	88.6 (54.4)	44.3 (71.0)	97.4 (70.1)	22.5 (21.6)
ALT, U/L	92.5 (72.0)	61.7 (52.8)	86.9 (66.0)	15.1 (26.2)
AP, U/L	403.3 (377.6)	223.3 (175.4)	345.5 (227.6)	38.7 (16.8)
γ-GT, U/L	262.3 (226.2)	231.9 (204.0)	349.2 (252.4)	18.6 (4.8)
Albumin (g/dl)	3.6 (0.9)	3.4 (2.4)	2.9 (1.1)	4.5 (2.3)
γ-globulin (g/dl)	1.7 (1.1)	1.7 (1.6)	1.5 (1.8)	1.1 (0.2)
AMA M2	157 (86%)	0 (0%)	0 (0%)	0 (0%)
Early histological stage (I/II)	119 (65%)	6 (60%)	11 (28%)	0
Advanced histological stage (III/IV)	58 (32%)	3 (30%)	5 (13%)	0
Ambiguous histological stage	5 (3%)	0	0	0

Data are presented as mean ± SD. Abbreviations: γ-GT, γ-glutamyl transpeptidase; ALT, alanine aminotransferase; AP, alkaline phosphatase; AST, aspartate aminotransferase. Normal value: bilirubin < 1.2 mg/dL; AST < 40 U/L; ALT < 40 U/L; AP < 115U/mg/dL; γ-GT < 50 U/L; albumin 3.5-5,5 g/dL, γ-globulin < 3 g/dL. Conversion factors to SI units are as follows: for bilirubin, 17.1; for AST, ALT, AP and γ-GT, 0.0167.

### Occurrence and Diagnostic Value of MMP-3

**Figure 1:**
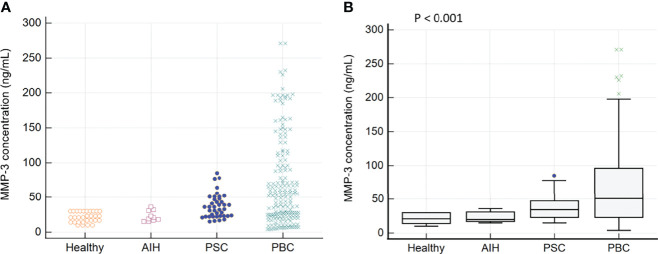
The MMP-3 concentration in sera of patients with PBC and control groups: **(A)** Distribution of MMP-3 in PBC patients and the control groups **(B)** The mean concentration of MMP-3 in each of the tested groups.

In the tested PBC patients, a higher concentration of MMP-3 was found in 112 out of 182 samples (61%). In the control group, among PSC and AIH patients, an increased concentration of MMP-3 was found in 22 out of 40 (55%) and 1 out of 10 (10%) patients, respectively. We did not observe an elevated concentration of MMP-3 in any of the healthy control sera. The concentration of MMP-3 in sera of PBC patients and control groups is presented in **Figure 1**.

**Figure 2:**
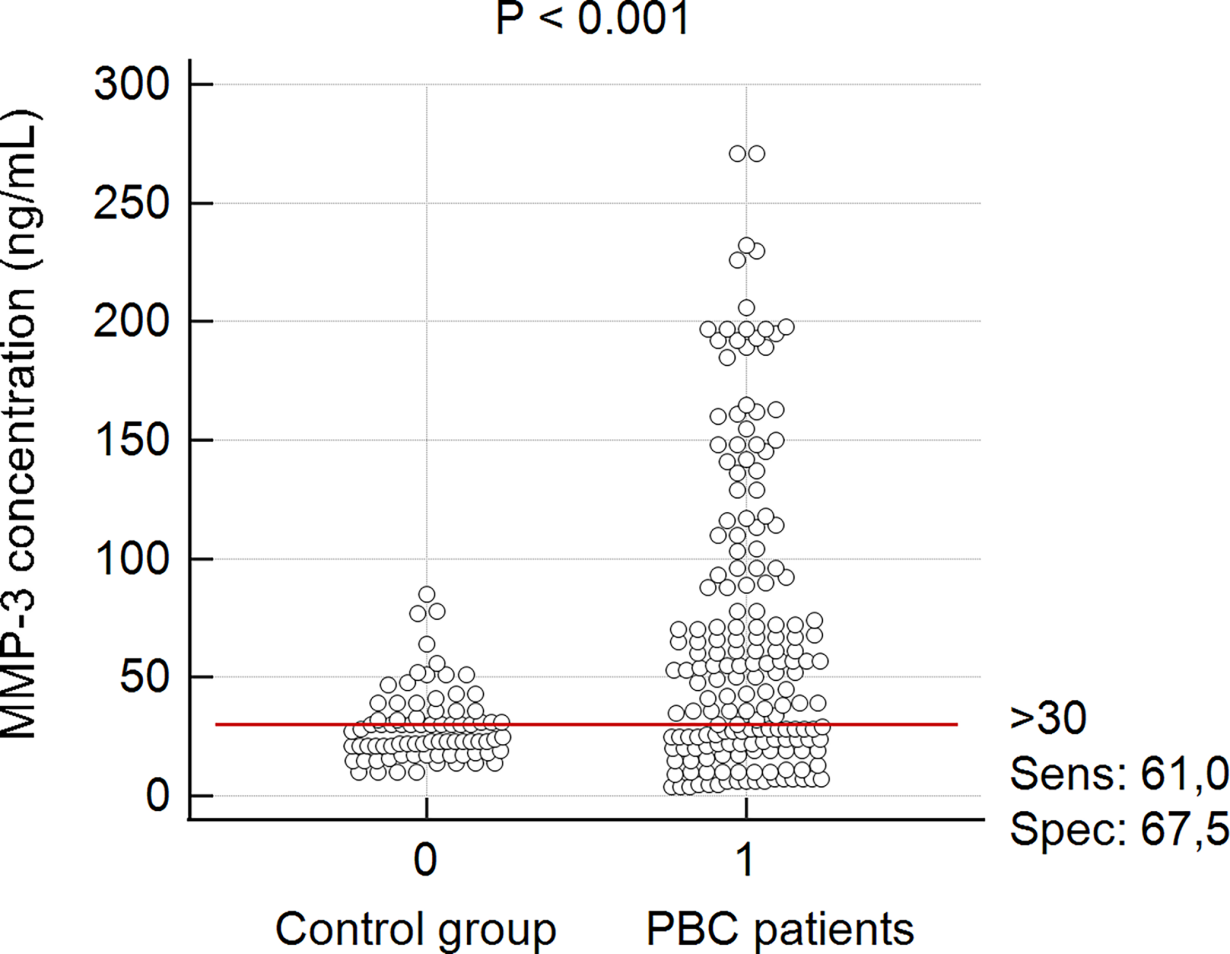
MMP-3 concentrations in accordance to the cut off value from manufacturer’s instructions. Control group – sera from patients with PSC, AIH and healthy donors.

There was a significant difference between the mean concentration of MMP-3 in the group of PBC patients and the healthy control group: 68.9 ± 62.6 ng/mL vs 21.3 ± 7.4 ng/mL, p < 0.0001. PBC subjects also had greater MMP-3 levels than AIH controls (68.9 ± 62.6 vs 22.7 ± 7.6 ng/mL, p = 0.022), PSC controls (68.9 ± 62.6 vs 37.2 ± 17.4 ng/mL, p = 0.002). In accordance to the manufacturer’s instructions, MMP-3 concentrations > 30 ng/mL were considered positive and for this cut-off value, the specificity and sensitivity were 67.5% and 61%, respectively (**Figure 2**).

**Figure 3:**
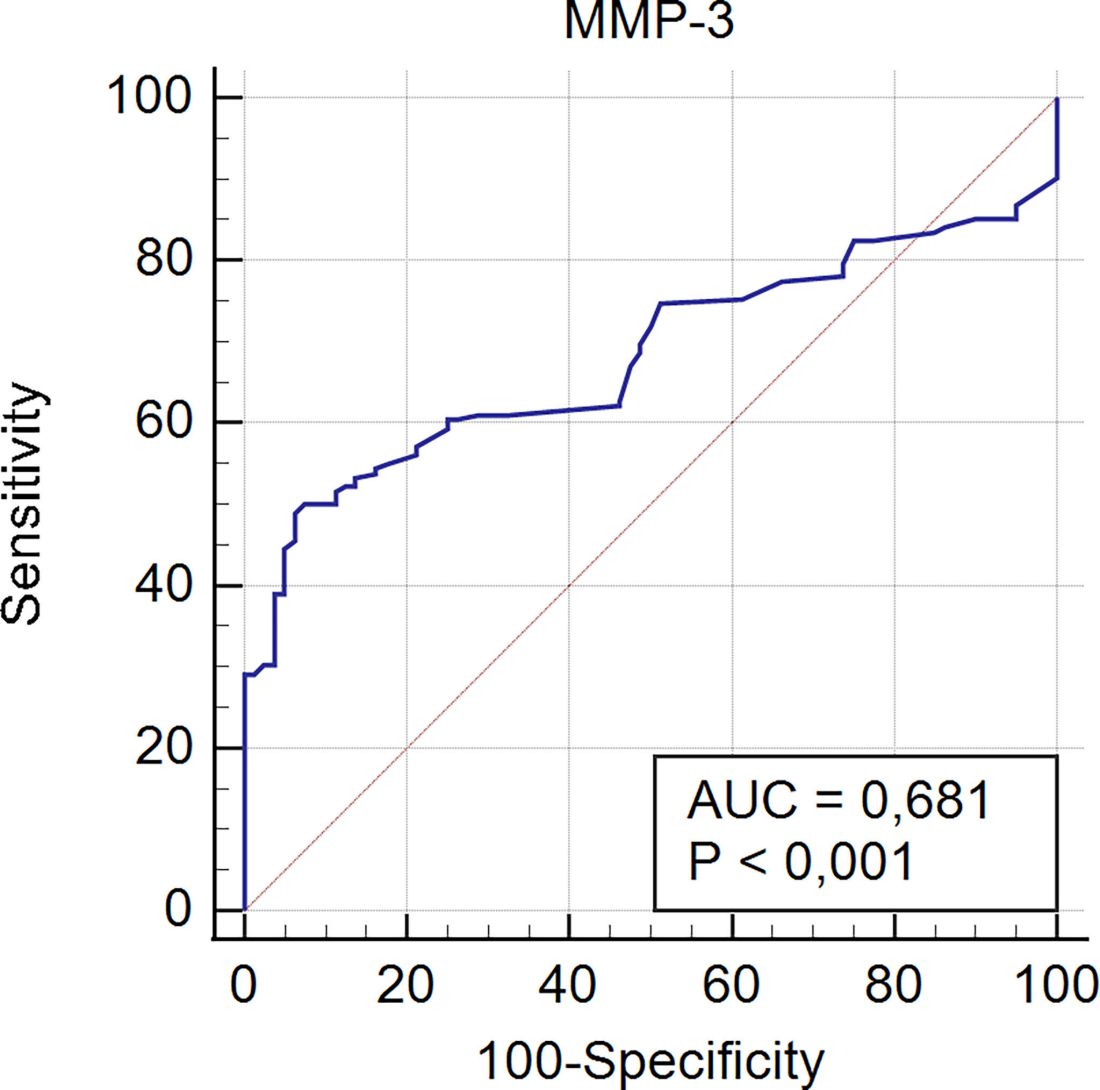
Receiver operating characteristic curve analysis for serological detection of MMP-3 in PBC patients.

Receiver operating characteristic curve analysis for serological detection of MMP-3 in PBC patients is presented in **Figure 3**.

### MMP-3 Concentration and PBC-Specific Antibodies

**Figure 4:**
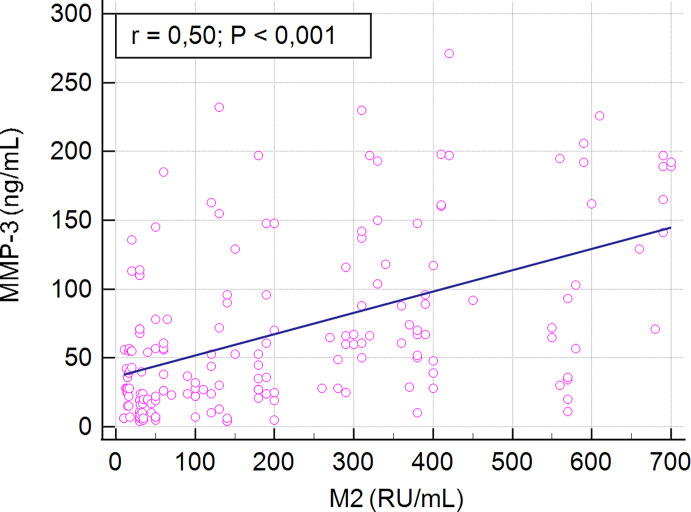
Correlation between serum MMP-3 concentration and AMA M2 levels in PBC patients.

We found a positive correlation between serum MMP-3 concentration and AMA M2 levels in PBC patients (r = 0.5, p < 0.001) (**Figure 4**).

**Figure 5:**
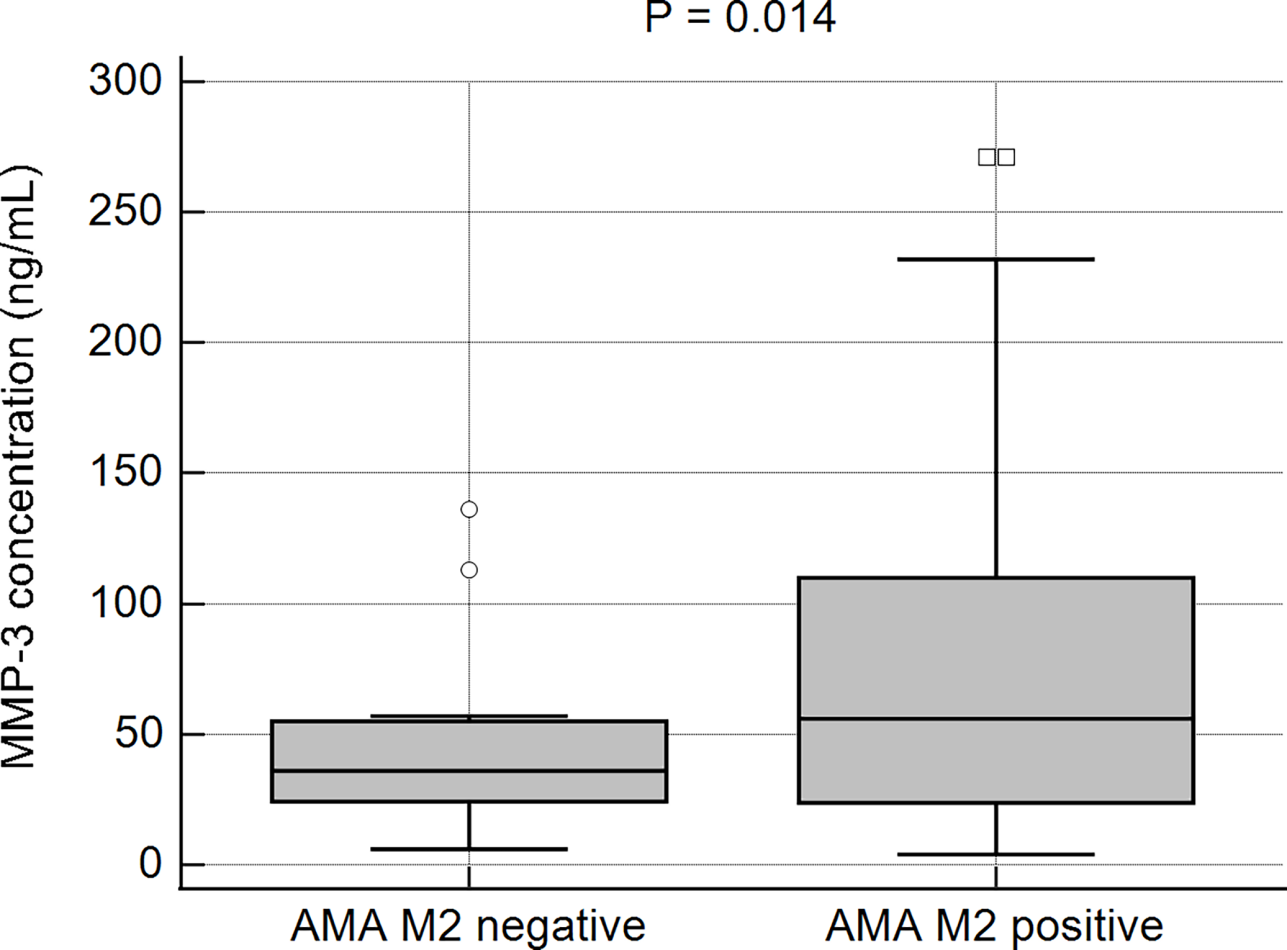
The MMP-3 concentrations in the AMA M2-positive and AMA M2-negative PBC patients.

The prevalence of patients with higher MMP-3 concentrations in the studied AMA type M2- positive and -negative populations was further evaluated. Higher MMP-3 concentrations were observed more frequently in AMA M2-positive (64%; 100/157) than in AMA M2-negative patients (48%; 12/25). This difference was not found to be statistically significant (p > 0.05). However, the mean concentration of MMP-3 in the AMA M2-positive patients was significantly higher than in the negative group: 73 ± 65.0 ng/mL vs 40 ± 30.1 ng/mL, p = 0.014 (**Figure 5**).

### Biochemical Features of PBC Patients According to the Level of MMP-3

**Figure 6:**
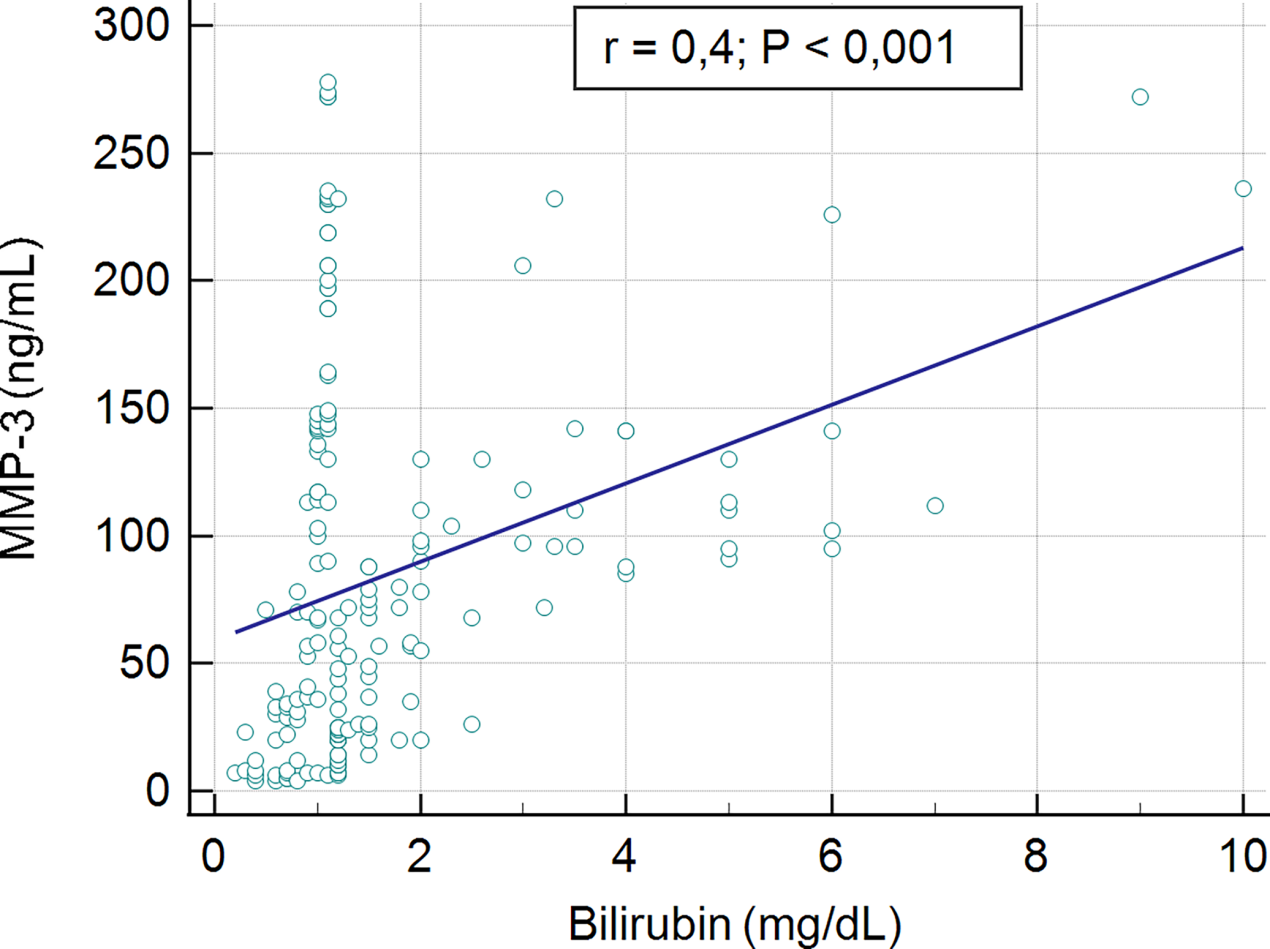
Correlation between serum MMP-3 concentration and level of bilirubin.

No association was found between the serum concentration of MMP-3 and biochemical markers such as AST, AP and γ-GT (r = 0.04, p > 0.05, r = 0.08, p > 0.05, r = 0.07, p > 0.05; respectively). A low, statistically significant correlation was shown between the serum concentration of MMP-3 and ALT level, r = 0,2, p = 0.04. The significant moderate correlation was presented for serum bilirubin level and MMP-3 concentration, r = 04, p < 0.001. The association between serum MMP-3 and bilirubin present **Figure 6**.

A comparison of groups of PBC patients who were positive and negative for MMP-3 showed that the symptoms of the disease began at the same age in each group of patients. The results of laboratory tests performed at the time of diagnosis were comparable in patients with normal and higher concentrations of MMP-3, with the exception of bilirubin and alanine aminotransferase (ALT) levels. Data from biochemical analyses performed at the time of diagnosis of 182 PBC patients, according to their MMP-3 status, are presented in **Table 2**.

**Table 2:** Data from biochemical analyses of 182 PBC patients and the MMP-3 status.

	MMP-3 concentration < 30 ng/mL N=70	MMP-3 concentration> 30 ng/mL N=112	*p-*value
**Bilirubin (n.v. < 1.1 mg/dl)**	0.9 (0.7)	2.1 (2.7)	< 0.001
**AST (n.v. < 37 IU/l)**	82.9 (59.8)	88.3 (53.3)	> 0,05
**ALT (n.v. < 65 IU/l)**	75.5 (54.4)	101.1 (90.1)	0.038
**AP (n.v. < 136 IU/l)**	375.5 (257.1)	407.1 (306.6)	> 0.05
**γ-GT (n.v. < 55 IU/l**)	256.5 (224.2)	265.2 (249.9)	> 0.05

AST, alanine aminotransferase; ALT, aspartate aminotransferase; AP, alkaline phosphatase; γ-GT, γ- glutamyl transpeptidase; n.v., normal value; data presented as mean (SD).

**Figure 7:**
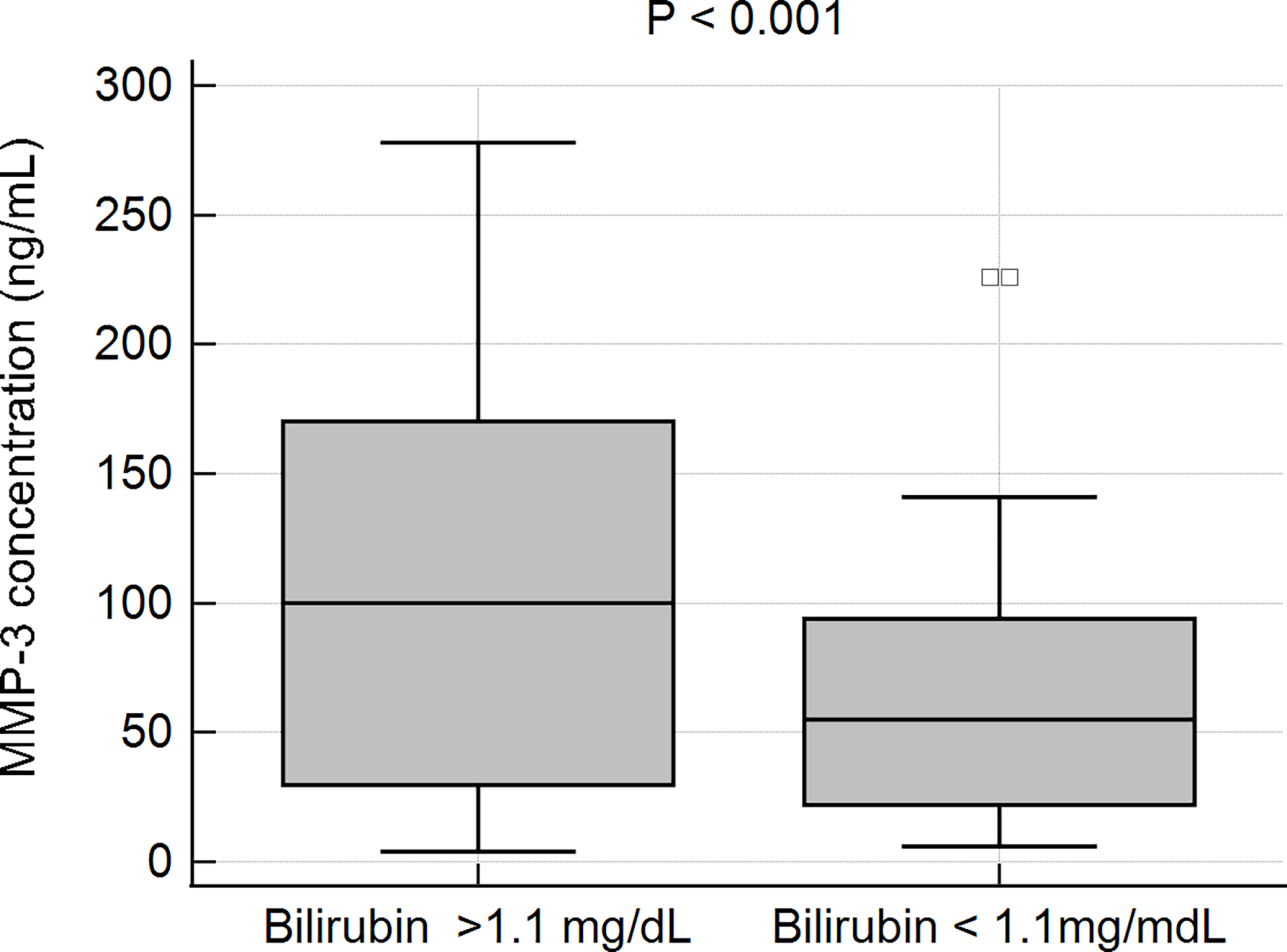
MMP-3 concentration and serum bilirubin in PBC patients.

In patients with elevated levels of bilirubin, the mean concentration of MMP-3 was observed to be 107.6 6 ± 85.8 ng/mL, while in patients with a normal bilirubin level the MMP-3 concentration was 61.6 ± 46.1 ng/mL. This difference was statistically very significant, p < 0.001 (**Figure 7**).

### Analysis of the Correlation Between Histological Parameters of PBC Patients and the Concentration of MMP-3

The serum MMP-3 concentrations were significantly correlated with the degree of liver fibrosis (r = 0.5, p<0.0001).

Patients with slight changes in liver tissue and patients with advanced fibrosis, according to Ludwig’s classification, were compared. More subjects with a higher concentration of MMP-3 were found in the group of patients with advanced fibrosis. The calculated OR (95% CI) for the histological score was 4.3 (2.0-9.2), p < 0.01. Among 119 PBC patients with early histological stages (I/II) of the disease, 63 (53%) had higher levels of MMP-3, while in the group of patients with advanced histological stages (III/IV), 48 out of 58 (83%) subjects were positive for MMP-3. This difference was statistically significant (p < 0.01).

**Figure 8:**
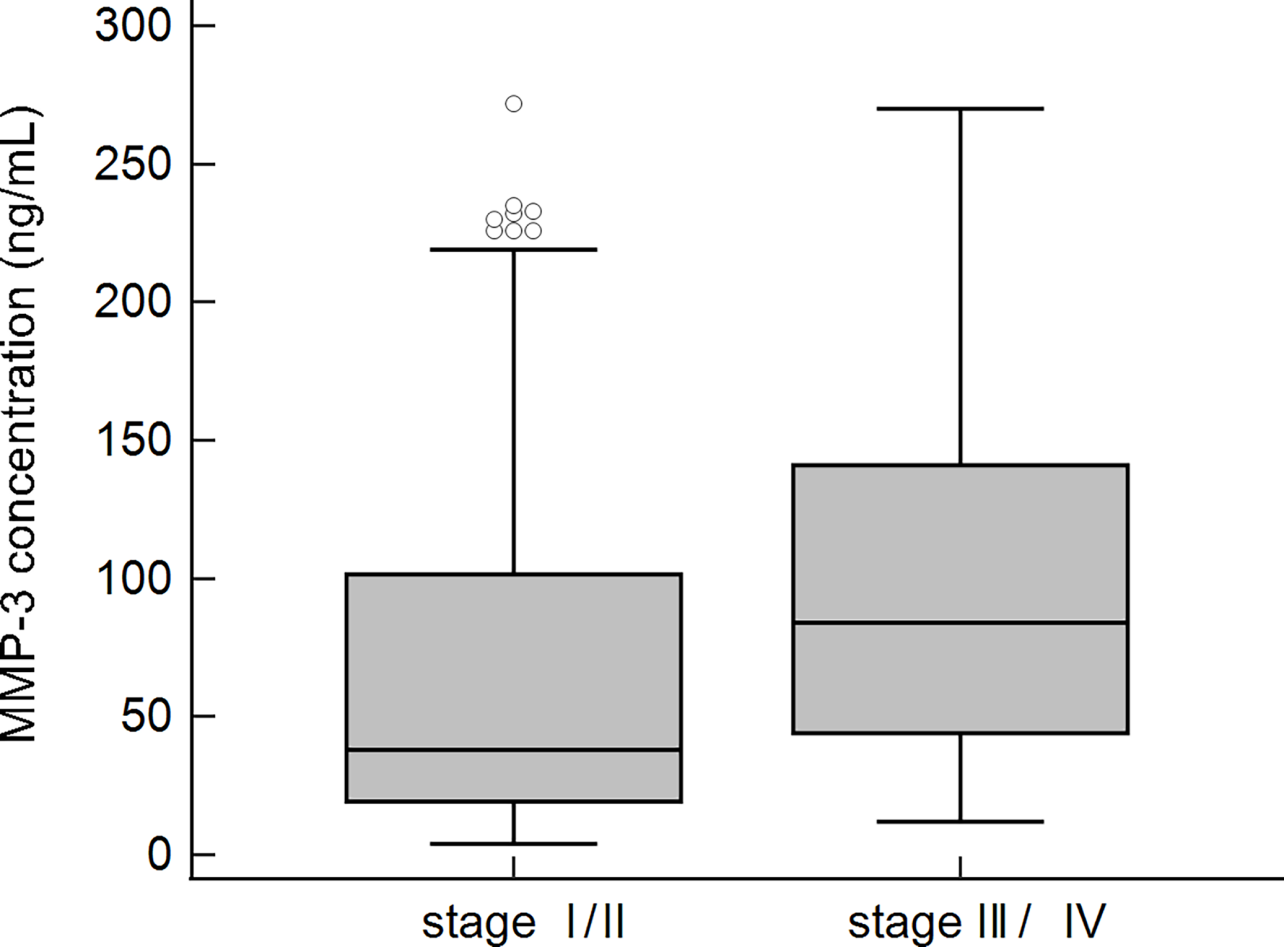
MMP-3 concentration in sera of PBC patients and the stage of fibrosis according to Ludwig’s classification.

The concentration of MMP-3 was also significantly higher in the group of patients with advanced fibrosis 103.1 ± 73.9 ng/mL vs 69.9 ± 68.7 ng/mL, p < 0.001 (**Figure 8**).

## Discussion

MMP-3 is associated with the occurrence and development of various diseases (53). Elevated serum MMP-3 levels have previously been reported in connective tissue disease: systemic lupus erythematosus (SLE) and rheumatoid arthritis (RA) (54–57) and also in myasthenia gravis (58). However, little is known about the role of MMP-3 in others autoimmune disorders. Therefore, in the presented study we aimed to determine MMP-3 concentrations in sera obtained from PBC patients.

Analyses of biochemical parameters and specific autoantibodies are standard procedures in the detection of PBC. Moreover, determination of markers of inflammation or fibrosis can be supportive for the diagnosis. We decided to study MMPs as they are involved in many distinct processes in the liver and play a critical role in the development of liver fibrosis. There is relatively little data in literature on the relationship between PBC and MMP-3. It has already been shown that MMP-3 is involved in liver inflammation, degradation of normal ECM, and release of cytokines that initiate macrophage and leukocyte infiltration. Our main aim was to assess whether MMP-3 is involved in liver progression and may be considered as a marker of fibrosis. We found significantly higher concentrations of MMP-3 in PBC patients in comparison to control subjects (healthy, AIH and PSC patients). PBC and PSC are both fibroinflammatory cholangiopathies, so it is interesting that PBC has higher levels of MMP-3 than PSC. The increase in MMP-3 in the liver and thus in the serum of patients may be related to the increased transforming growth factor (TGF) -β1 in the fibrotic liver. Expression of TGF-β1 is increased in patients with chronic liver disease. MMP-3 expression may also be associated with an increased amount of type IV collagen. Initial damage to cholangiocytes in autoimmune liver diseases is related to the innate immune response. Cytokines and metalloproteinases may be involved in the process of fibrosis both at PBC and at PSC. However, the mechanism of the fibrosis process involving metalloproteinases is not fully understood. Perhaps higher MMP-3 level in PBC is related to the location of the disease process. PBC targets the small bile ducts, PSC targets the large bile ducts. Despite sharing common symptoms (such as itching and fatigue) and having comparable acronyms, PSC and PBC are distinct entities and exhibit important differences, which include the site of tissue damage within the liver, associations with inflammatory bowel disease (IBD, which includes ulcerative colitis and Crohn’s disease) response to treatment, risks of disease progression and cancer. However, the two diseases are not the same, despite sharing certain similar characteristics and symptoms. Each condition creates different needs among patients, and each requires different treatments and monitoring. Some PSC patients suffer from concomitant inflammatory bowel diseases, but also from infectious diseases of the biliary tract. Individuals with PSC can occasionally develop abdominal pain and fever, which may suggest infection of the bile ducts called bacterial cholangitis. Although the latter can be treated with antibiotics, no currently known treatment has been shown to slow the progression or cure PSC. Perhaps treatment with antibiotics affects the level of MMP-3. The use of tetracycline-like antibiotics, which inhibit MMPs, were previously approved for treating infection (59). The reason for the increased activity of MMP-3 and the relationship of MMP-3 to liver fibrosis is not entirely clear. Higher MMP-3 activity in pathological conditions may be associated with liver fibrosis through the cell-matrix interaction mechanism. Changing the matrix composition results in the activation of cells in the liver, leading to proliferation and fibrogenesis. These processes need not be identical for the PBC and the PSC.

Very high MMP-3 concentrations have previously been reported in SLE and RA patients (above 250 ng/mL and 180 ng/mL, respectively), which are higher than in our PBC patients. As shown by Honsawek et al., who studied diseases of the bile ducts, serum MMP-3 levels in biliary atresia (BA) patients were markedly higher than those in healthy controls. Untreated BA patients were more likely to develop severe liver fibrosis, biliary cirrhosis, and eventually die before they were 2 years old (60). Although the diseases affect different age groups, it can be considered that the pathophysiological mechanism may be similar. Honsawek et al. revealed that BA patients with high ALT had increased concentrations of serum MMP-3, as compared to those with normal ALT. In our study, a correlation between the presence of higher concentrations of MMP-3, and higher levels of bilirubin and ALT was also found. It has been known that high serum ALT is a specific indicator for liver injury. This is important as it has been suggested that bilirubin levels predict liver transplantation or death in PBC patients and are used in selection of treatment strategies (61, 62). The group of PBC patients with an elevated level of bilirubin presented a significantly higher concentration of MMP-3. No association was found between the serum concentration of MMP-3 and biochemical markers such as AST, AP.

We also evaluated the concentration of MMP-3 in the group of PBC patients with AMA type M2 antibodies, because of their high sensitivity and specificity for diagnosis of PBC. We found that the increased concentration of MMP-3 in PBC was determined more often in the AMA M2 positive group. We found a positive correlation between serum MMP-3 concentration and AMA M2 levels in PBC patients

Finally, we studied MMP-3 concentrations in patients with both very slight changes in liver tissue and with advanced fibrosis, I/II stages and III/IV stages, respectively, according to Ludwig’s classification. In the groups with advanced fibrosis we found more patients with increased concentrations of MMP-3. The calculated OR was above 4, when compared to patients with slight changes in liver tissue. The concentration of MMP-3 was also significantly enhanced in the group of patients with advanced fibrosis.

Previous studies have presented correlations between increased MMP-3 and production of endostatin (by collagen XVIII) (63), which is connected with lung epithelial cell apoptosis (64) and thus promotes fibrosis. Perhaps a similar scenario may occur in the case of liver fibrosis, as elevated levels of MMP-3 were found in the sera of these patients. Our results may suggest that a high serum concentration of MMP-3 can be related to the degree of liver fibrosis.

We propose that the concentration of MMP-3 in sera, in connection with the level of bilirubin and presence of specific antibodies could be used as a non-invasive biomarker to determine the possible severity of liver disease.

We can try to reflect if MMP-3 could be a therapeutic target? The process of liver fibrosis may be reversible and the removal of damaging factors may be an effective treatment option, for example remodeling the ECM, affecting the activity of MMPs. A therapeutic target in the case of fibrosis could be the regulation of ECM-degrading enzymes. Some studies show that interfering with the activity or expression of MMPs reduces fibrosis. Giannandrea and Parks present fibrosis treatment outcomes for various MMPs (25). In turn, Hemman and colleagues note that MMP expression is elevated in both the early and advanced stages of fibrosis, prior to scar tissue accumulation, and that levels decline after treatment. The authors discuss MMPs for the treatment of fibrosis (65). Our study shows that the concentration of MMP-3 also increases in the early stage of fibrosis. Perhaps it is the inhibition of MMP-3 that could play an important role in the treatment that slows down the fibromyalysis process. The relationship between fibrosis and MMPs like MMP-1, MMP-3, MMP-7, MMP-9, MMP-13 and MMP-19, in macrophages and neutrophils was increased for example in BALF. These infiltrating macrophages and neutrophils are associated with the inflammatory response and are essential in pulmonary fibrosis (66, 67). Perhaps we could observe similar processes concerning the liver. Hong-Meng Chuang presents inhibitors of MMPs, such as PD166793 hydrate S-2- (4’-bromobiphenyl-4-sulfonylamino) -3-methylbutyric acid), effective in animal models of pulmonary, liver and myocardial fibrosis (68). However, most of them are drugs used in animal models and must be tested in preclinical studies before they can be used in patients.

In conclusion, we found a significantly higher MMP-3 concentration in PBC patients than in healthy controls. A positive correlation between higher MMP-3 levels, and presence of specific AMA type M2 autoantibodies and increased bilirubin concentration suggests that MMP-3 can be associated with a rapidly evolving disease of the liver, including hepatic fibrosis in PBC patients.

## Data Availability Statement

The raw data supporting the conclusions of this article will be made available by the authors, without undue reservation.

## Ethics Statement

The studies involving human participants were reviewed and approved by Ethical Committee of the Centre of Postgraduate Medical Education, Warsaw (approval number 71/PB/2019). The patients/participants provided their written informed consent to participate in this study.

## Author Contributions

AB obtained the funding, was responsible for conception, design, and coordination of the study, performed the serum assays, performed the statistical analysis, elaborating the table and figure, analysed and interpreted the data, drafted the manuscript and submitted the manuscript. AH evaluated patients, collected the clinical samples and data, analysed and interpreted the data. All authors have given final approval of the version to be published.

## Funding

The study was supported by grant: 501-1-025-01-21 from the Centre of Postgraduate Medical Education, Warsaw, Poland.

## Conflict of Interest

The authors declare that the research was conducted in the absence of any commercial or financial relationships that could be construed as a potential conflict of interest.

## Publisher’s Note

All claims expressed in this article are solely those of the authors and do not necessarily represent those of their affiliated organizations, or those of the publisher, the editors and the reviewers. Any product that may be evaluated in this article, or claim that may be made by its manufacturer, is not guaranteed or endorsed by the publisher.
